# Efficacy and biomechanical effects of the powered lower-limbs exoskeletons Keeogo in adults with neuromuscular diseases

**DOI:** 10.1186/s12984-025-01867-7

**Published:** 2026-01-17

**Authors:** R. Feigean, C. Afroun-Roca, C. Guerrini, J. Souchu, F. Fer, G. Bassez, O. Benveniste, J. Y. Hogrel, D. Bachasson

**Affiliations:** 1 Institute of Myology, Neuromuscular Investigation Center, Neuromuscular Physiology and Evaluation Laboratory, Paris, France; 2 Institute of Myology, MyoData Team, Paris, France; 3 Neuromuscular Reference Center, Pitié-Salpêtrière Hospital, Assistance Publique Hôpitaux de Paris, Paris, France; 4 Sorbonne Université, INSERM, UMRS1158 Neurophysiologie Respiratoire Expérimentale et Clinique, Paris, France

**Keywords:** Robotized exoskeleton, Neuromuscular diseases, Assistive devices, Biomechanics, Gait, Muscle weakness, Functional impairments

## Abstract

**Background:**

Neuromuscular diseases (NMD) result in progressive muscle weakness, significantly impairing functional abilities and quality of life. Assistive devices like the Keeogo, a lightweight, non-self-supported powered exoskeletons have the potential to enhance mobility and independence in NMD. This study seeks to fill the gap in our understanding of how these devices impact mobility and function in this specific population.

**Methods:**

We investigated efficacy, perceptions and biomechanical effects of the powered exoskeleton Keeogo in adults with NMD during a 2-min walk test (2MWT), 10-m walk test (10mWT), 30-s sit-to-stand test (STS30), and postural stability test (SWAY). Patient’s perception of stability, exertion, dyspnea and pain, muscle strength, gait parameters, joint kinematics, and muscle activation were evaluated.

**Results:**

Knee extensor strength was 45.5 ± 63.5 Nm, corresponding to 27.6 ± 42.9% of predicted value based on normative data. Walking performance during 2MWT and 10mWT were significantly reduced while wearing the Keeogo, with decreased gait speed, stride length, and cadence. Hip flexion–extension range of motion was reduced and g*astrocnemius medialis,* r*ectus femoris,* and v*astus lateralis* muscle activation were diminished when using the device. Major contributors to reduced gait performance when using the device identified using LASSO model was as follows: knee extension and ankle dorsiflexion strength, cadence, ankle angle at toe-off, and late hip extension and ankle dorsiflexion peak angles. The *gluteus maximus* muscle activation was lower during chair tests when using the Keeogo. No changes in static postural stability were observed.

**Conclusions:**

The Keeogo exoskeleton, while not improving static postural stability, adversely affected dynamic walking and transfer performance and kinematics the studied groups of adults with NMD, proportionally to the level of weakness and functional impairments. However, muscle loading was lessened in several muscle groups, emphasizing the potential of the device for alleviating muscle load. These findings highlight the need for customized exoskeleton hardware and software design to optimize assistance to the severity and distribution of muscle weakness.

**Supplementary Information:**

The online version contains supplementary material available at 10.1186/s12984-025-01867-7.

## Background

Neuromuscular diseases (NMD), such as muscular dystrophies, inflammatory myopathies, or congenital myopathies, are characterized by progressive muscle weakness. The extent of functional impairment is primarily determined by the severity and distribution of this weakness and is associated with reduced mobility, autonomy, and quality of life [1, 2]. Conversely, a residual level of muscle strength may be retained over the years allowing ambulation and primary transfers, even at advanced stages [2]. These abilities rely on compensatory strategies that impose high mechanical loads on joints and muscles, increasing fatigue and fall risk [3].

Recent advances in wearable robotics have led to the development of lightweight powered lower-limb devices designed to enhance gait and transfer performance in various populations [4, 5]. Their architecture varies widely across assisted joints (hip, knee, ankle) and weight distribution systems, which strongly influence usability and functional outcomes. This variability poses particular challenges for individuals with NMD, whose weakness and balance impairments are highly heterogeneous.

In this context, the Keeogo (B-Temia Inc., Quebec, Canada) has emerged as a promising powered device providing assistance during gait and sitting transfers [6, 7]. It provides powered assistance for knee flexion and extension [7], is lightweight (< 10 kg), non–self-supported (i.e., users bear its weight), and assists during walking and sitting transitions when triggered by the user’s movement initiation. Importantly, this exoskeleton was originally intended to assist lower-limb weakness or instability (e.g. knee osteoarthritis, stroke) but was not specifically designed for NMD. Previous studies reported that some Keeogo users with neurological diseases (e.g. multiple sclerosis, stroke) showed improved performance during 30-s sit-to-stand test (STS30) and stair test [8]. However, performance during the 6-min walk test (6MWT) was often reduced (10 of 13 participants). The added weight of exoskeletons in individuals with significant muscle weakness may counteract potential benefits, raising doubts about their effectiveness. Moreover, the compensatory strategies adopted by participants introduce additional uncertainties regarding the device’s benefits [9], which remain unexplored in NMD.

Previous studies in NMD have mainly focused on performance (e.g., distance and timed tests) without investigating the neuromuscular and biomechanical mechanisms underlying these limitations. Yet, reductions in gait speed and cadence reflect compensatory adjustments to lower-limb weakness and correlate with disease severity and fatigability [10, 11]. Recent work revealed greater muscle activation in knee flexors and extensors in NMD, reflecting compensatory strategies to maintain stability and propulsion despite muscle weakness [12]. In Late-onset Pompe disease, single-support duration, hip abductor strength, and altered knee flexion at foot strike were identified as major determinants of locomotor performance [13], underlining the value of a multimodal approach combining force and kinematic evaluation. Building upon these findings , we recently developed an integrated framework combining measurements of muscle strength, joint kinematics, muscle activation, and perceptions [12]. This approach reveals key determinants of gait performance, providing a methodological foundation for evaluating the effects of assistive devices such as the Keeogo in NMD.

Therefore, this study aimed to assess the effects of the Keeogo on performance (time or repeated transfers), muscle activation, joint kinematics, and perceived exertion in adults with NMD. We hypothesized that the Keeogo would not improve walking performance but would modify kinematic and neuromuscular patterns and reduce perceived effort. We further hypothesized that individuals with milder weakness would derive greater benefit. Moreover, we applied multimodal approach to identify determinants of individual responses to assistance, thereby bridging functional and biomechanical findings. This integrated approach offers new insight into how assistive devices interact with the unique biomechanical constraints of NMD.

## Materials and methods

### Participants

Individuals aged between 18 and 70 years diagnosed with muscular dystrophies, inflammatory myopathies, or congenital myopathies were included. Participants were required to be able to walk independently for two minutes in a corridor and perform at least three sit-to-stand in thirty seconds without assistance from other people. Technical aids such as canes, crutches, light knee and ankle orthoses were permitted. Exclusion criteria comprised individuals using rigid knee braces, those with unstable cardiomyopathy, orthostatic hypotension, balance disorders with non-neuromuscular causes, or recent trauma (within the last six months), severe osteoporosis, severe vascular disorders of the lower limbs. All participants provided informed written consent. The study conformed to the Declaration of the Helsinki and was approved by the ethics committee (ID RCB: 2020-A02160-39, Clinical Trials: NCT05200702).

### Experimental protocol

Participants attended three visits each spaced 2 days to 2 weeks apart. During the first visit, participants were familiarized with the Keeogo. Installation involved fitting the padding size to each participant. Then the assistive torque and knee angle range of motion were adjusted to the participant’s specific needs during the gait cycle, following the manufacturer’s instructions. Each familiarization session included two parts: identifying preferred settings, particularly during the first visit, and training with those settings. Subsequent visits focused on training, with adjustments made as needed. During the second and third visits, strength assessments were performed using an isokinetic dynamometer (Biodex System 4-Pro, Biodex, Inc., Shirley, New York) as previously described [14]. Participants performed three repetitions of isometric maximum voluntary contractions (MVC) for hip and knee flexion and extension, and ankle plantar- and dorsi-flexion. Throughout the MVCs, surface electromyography (EMG) was used to assess maximal muscle activation required to normalize EMG signals during functional tasks (see “recordings during tests” section). Then, participants performed successively a STS30, three repetitions of static postural stability test (SWAY), two repetitions of a 10-m walk test (10mWT) and a 2-min walk test (2MWT). These tests were conducted without the device during the second visit and with the device during the third visit. Recorded variables were as follows: number of repetitions (STS30), time duration (10mWT) and distance covered (2MWT), perceptions, and gait parameters using inertial measurement units (IMUs). At the end of the second visit, participants performed a second familiarization session with the device.

### Exoskeleton device

The Keeogo device is a powered lightweight lower limb, and non-self-supported exoskeleton assisting in knee flexion and extension during the swing phase of the gait cycle. It is a user-initiated device, and the severity of an individual’s disability affects the functionality of the system. It comprises bilateral motors to assist both left and right knees, a pelvis belt, and a chariot system for suspending the device. Additionally, thigh and shank padding from the device are attached to the user. The controller can recognize standing, walking, stair ascent and descent, chair rising/siting, and other locomotor tasks. The knee motors provide assistance for right and left legs separately during the swing phase during gait while the hip motion of the device is unassisted. The shin cuffs can accommodate most ankle–foot orthoses, and the device can be used alongside other assistive aids such as canes or crutches. The total weight of the Keeogo, including the battery pack, is 5.4 kg. During the familiarization sessions, the Keeogo device was adjusted for each participant by setting the thigh length, waist belt, and leg cuffs to achieve optimal suspension and alignment. The device was initially set to “passive” mode, allowing participants to move freely while adjustments were made as needed. Then, the device’s assistance was adjusted progressively to deliver the maximum level of assistance (N.m), ensuring the settings remained acceptable for the participant. Constant feedback on the perceived assistance and knee flexion and extension range of motion assistance was used to individually optimize the settings aiming to deliver the maximal acceptable assistance level, following manufacturer guidelines for proper setup [7].

### Recordings during tests

The STS30 was performed on a chair with armrests that were allowed to be used. Participants were asked to repeat sit-to-stand and stand-to-sit transitions as many times as they could in 30 s [15]. Postural SWAY test was performed eyes open pointing a mark on the wall and on a firm surface for 30 s. Participants were asked to stay as still as possible during the whole trial [16]. Common spatio-temporal parameters during postural SWAY were measured from IMUs Opal V2C^©^ system (APDM, Portland, USA): 95% ellipse axis 1 and 2 (m.s^−2^), mean velocity (m.s^−1^), root mean square sway (m.s^−2^), and sway area (m^2^.s^−4^). The 10mWT was performed according to guidelines [17]. The 2MWT was performed on a twenty-five-meter track [18]. Participants were asked to cover the longest distance possible, self-managing their efforts. Participants had at least three minutes resting period between each test and trial. Verbal analog scale (VAS) was used to measure participants’ perceptions of stability, exertion, dyspnea and pain [19]. Participants were asked to provide a whole number between 0 and 10, where “0” represented the minimum conceivable and “10” the maximum conceivable. Perceptions using VAS were collected after each trial. At the end of the third visit, participants answered two validated questionnaires: the modified Nordic Musculoskeletal Questionnaire (NMQ) and the System Usability Scale (SUS) questionnaire [20, 21]. The NMQ was then reported on a scale from 0 as “no discomfort” to 10 as “extreme discomfort”. The SUS is divided in 5 categories from 0 as “worst usability” to 10 as “best imaginable usability”. Surface EMG activity was collected bilaterally with wireless surface electrodes (Trigno quattro-sensor or Trigno mini-sensor, Delsys Inc., Boston, USA) over *gluteus maximus* (Gmax), *biceps femoris* (BF), *vastus lateralis* (VL), *rectus femoris* (RF) and *gastrocnemius medialis* (GM) according to SENIAM recommendations [22]. For each muscle, electrodes were placed over muscles bellies and aligned along the orientation of the muscle fascicles [22]. Tapes attached electrodes to the skin to limit movement artifacts and prevent detachment. The EMG signals were amplified, digitized at 2000 Hz, recorded in LabChart PROV8.1.25 software (AD Instruments, Bella Vista, Australia) and band-pass filtered (20 to 500 Hz). Hip, knee and ankle joint kinematic and spatio-temporal parameters data was collected using eleven IMUs Opal V2C^®^ System over sternum, lumbar, thighs, calves, and feet according to manufacturer’s instructions. IMUs signals were recorded in Moveo Explorer software (APDM, Portland, USA), amplified, and digitized at 128 Hz. Each test and trial were pre-programmed and included a still period of three seconds before the start for IMU calibration.

### Data analysis

All data analysis was performed using the R environment. The highest strength values from the MVCs trials for each joint movement and sides were retrieved. The percentage of predicted muscle strength (MVC_pred_) was computed using previously published equations [14, 23]. Percentage of predicted distance for the 2MWT (2MWT_pred_) was computed using previously published equations [18]. For EMG data analysis, all signals were band-pass filtered using a fourth-order Butterworth filter (20–500 Hz), then full wave rectified, and low-pass filtered at 12 Hz to obtain the EMG envelope. The peak amplitude of the MVC envelope was identified as EMGmax, and gait cycle EMG envelopes were normalized to this reference. The following features were computed from the normalized EMG signal during stance and swing phases of each gait cycle and during sit-to-stand and stand-to-sit phases of each repetition for each recorded muscle: average peak value (EMG_peak_), average area under the curve as an index of muscle work (EMG_AUC_). During 2MWT, average and standard deviation of gait cycle percentage of EMG_peak_ occurrence (EMG_avg_ and EMG_SD_, respectively) were computed. The shift in the mean and median power frequency between the last tenth and the first tenth cycles of the 2MWT, was computed from the band-passed EMG signal to estimate induced muscle fatigue (EMG_meanPF_ and EMG_medianPF_, respectively) [24]. Kinematic/kinetic, joints range of motion and gait parameters were recorded and processed in the Moveo Explorer software (APDM). Joint angle values during 2MWT and STS30 were low-pass filtered using a second-order butter-worth filter (3.5 Hz) [25]. Gait cycles were normalized using the Moveo Explorer software’s automatic detection of heel strike and toe-off events, and time was expressed from 0 to 100% of the gait cycle. To ensure accuracy and reproducibility, all automatically detected cycles were visually inspected before further analysis. Regarding the STS30 cycle and phase detection, we used hip flexion signals involved the following steps: correction of extreme negative values, smoothing of the signal’s derivative to identify critical points, application of quantile-based thresholds to distinguish significant peaks, and identification of cycles by locating minima and maxima. Joint angles for each joint during each phase were averaged, including both stance and swing phases for 2MWT and sit-to-stand and stand-to-sit phases for STS30. The following features were computed: average joint range of motion (JA_RoM_), average and standard deviation of the percentage of the phase corresponding to the occurrence of peak joint angle (JA_avg_ and JA_SD_, respectively).

### Statistics

Statistical analyses were performed in the R environment. Normality of the data was tested using the Shapiro–Wilk test and visual assessments. As all variables were non-normally distributed, non-parametric tests such as the Wilcoxon rank-sum test were used to evaluate whether performance differs between the condition without and with the use of the Keeogo. Only participants with valid data for both conditions were included in the statistical comparisons. Effect sizes were computed using Cohen’s *d*. JA and EMG statistical analysis were averaged across sides during 2MWT and STS30 tests. The shape of EMG and JA signals over time were tested between conditions using statistical parametric mapping with a two-way repeated-measures ANOVA [26]. A Bonferroni correction was applied to address multiple comparisons bias. This analysis compared signal values (EMG or JA) at each percentage of the gait or STS30 cycles, with and without the use of Keeogo. Subsequently, time blocks were created to summarize occurrences of significant differences between signal shapes. Potential relationships between variables were assessed using Pearson’s correlation coefficients. A penalized Least Absolute Shrinkage and Selection Operator (LASSO) regression model was employed to identify key determinants of the predicted 2MWT distance measured under both conditions. Features from both assisted and unassisted conditions were included to identify the ones associated with the reduction in gait performance. Conversely, a separate LASSO model was developed to predict the change in sit-to-stand repetition count during STS30 between assisted and unassisted conditions and based on features measured during the unassisted condition (visit 2). This approach allowed us to integrate multiple correlated predictors into a single model and to determine their relative importance in explaining performance reduction (2MWT) or improvement (STS30) with assistance. For the LASSO predictive model of 2MWT and STS30, each corresponding dataset was randomly split into 80% training and 20% validation subsets. Each participant’s data was assigned to a single subset per iteration, ensuring that data from each participant was used only in one of the subsets at a time. The process was repeated 1000 times to ensure robustness. During each iteration, the variables selected in the predictive equation were recorded, and the model’s performance for both 2MWT and STS30 was evaluated using root mean squared error (RMSE), mean squared error (MSE), mean absolute error (MAE), and the coefficient of determination (R^2^), all calculated on prediction errors from the test dataset. The LASSO model was specifically chosen because it penalizes coefficients, effectively reducing the impact of correlated variables by shrinking some of them to zero, even if they may be good candidates. This regularization approach allows the model to select the most relevant predictors while mitigating the risk of overfitting caused by multicollinearity [27–29]. This iterative process allowed for the identification of contributing variables, even in the presence of potential collinearity, providing a partially explanatory model. We opted for 1000 iterations instead of evaluating all possible combinations to balance computational efficiency with robust variable selection, as exploring every combination would have been computationally prohibitive. To identify the top 500 models, we used the following criterion: models with the lowest prediction errors, based on the MSE calculated at each iteration on the test data, were selected. Specifically, models whose MSE ranked within the lowest 50% were retained for further analysis. This 50% threshold was determined after visual inspection of the distribution of prediction errors, which revealed a significant performance drop beyond this point. The variables selected in more than 50% for 2MWT and 70% for STS30 (due to lower dataset) of these top 500 models were retained for a second modeling cycle. These thresholds for variable selection were determined empirically for both tests, as the distribution of selection frequencies showed a marked decline around this value in the most performant models. Once the variables were identified, we assessed overfitting and estimated the variance in performance metrics by repeating this process with the selected variables. In each iteration, the prediction errors (RMSE, MSE, MAE, and R^2^) were calculated on the test dataset, to quantify performance variance and evaluate the robustness of the predictions. The means and standard deviations of the performance indicators were computed based on the distributions of the recorded metrics from each iteration. The estimation of the regularization parameter λ was performed only once at the beginning of each of the two 1000-iteration cycles, using the entire dataset and a tenfold cross-validation process. This optimal λ was then applied in each iteration of the LASSO model. At the end of all iterations, a final model was estimated using the entire dataset, with λ re-estimated similarly, but limited to the selected variables, to obtain the final coefficients and assess the overall performance of the model. This method enabled the prediction of STS30 performance improvements with the Keeogo and the identification of key determinants underlying the reduced 2MWT distance compared with the unassisted condition.

## Results

### Population

**Fig. 1:**
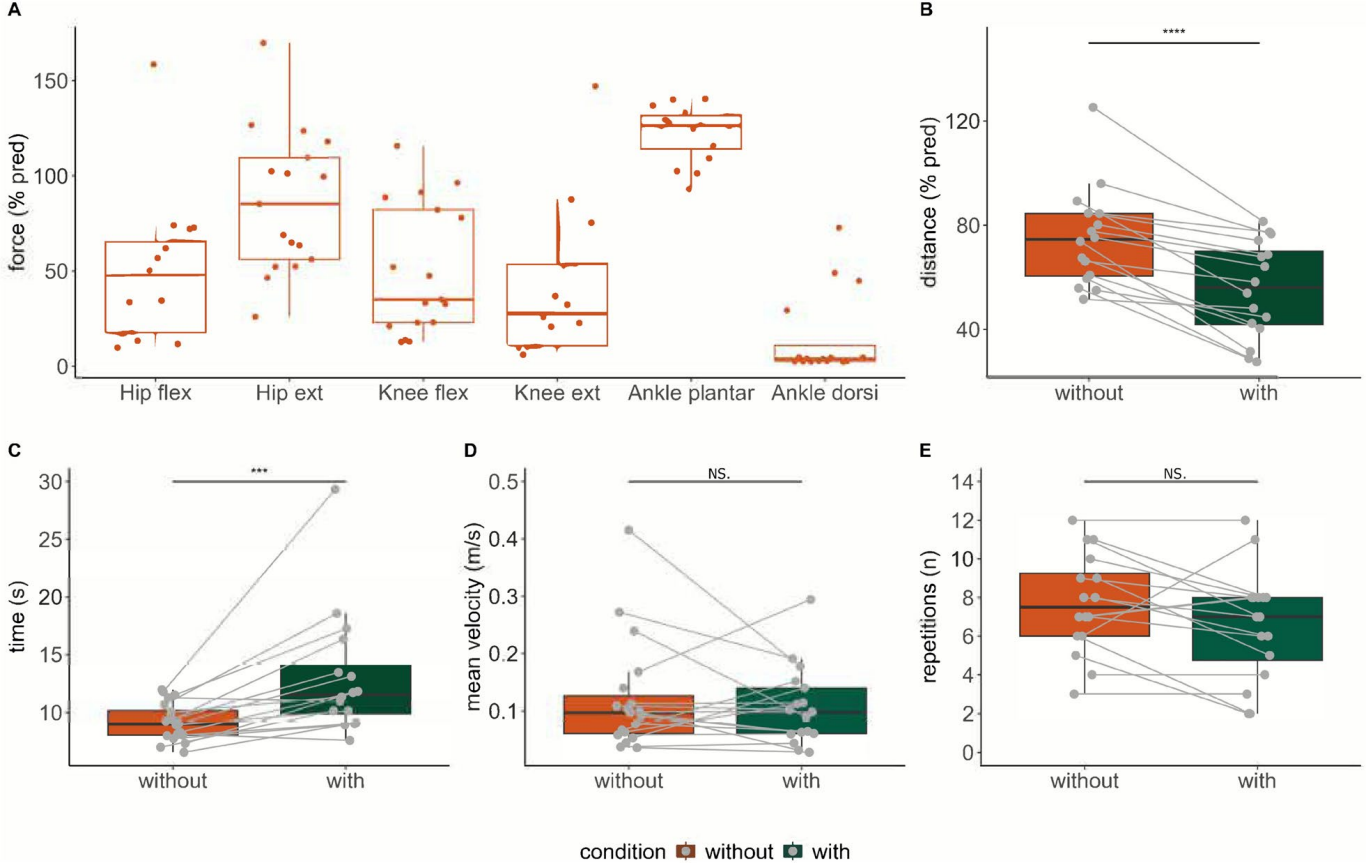
Maximal predicted lower-limb muscle strength (**A**). 2-min walk test predicted distance (**B**), 10-m walk test time duration (**C**), postural sway velocity (**D**) and 30-s sit-to-stand number of repetitions (**E**) without and with the use of the Keeogo. All variables are displayed as boxes and whiskers plots, median (thick middle line), interquartile range (end of box), and ranges between maximal and minimal value (vertical lines) are presented. Each participant is represented by a point in both the with and without exoskeleton conditions, connected by a line. ***, p < 0.001; ****, p < 0.0001. *Hip flex* hip flexion, *Hip ext* hip extension, *Knee flex* knee flexion, *Knee ext* knee extension, *Plantar flex* plantar-flexion, Dorsi flex dorsi-flexion

A total of 23 participants were included: 9 women, 14 men, age: 51.8 ± 12.5 years, body mass: 76.6 ± 13.8 kg, height: 1.8 ± 0.1 cm, BMI: 25.0 ± 5.2; 13 with muscular dystrophies (Fascio scapulohumeral dystrophy, Becker muscular dystrophy, myotonic dystrophy, limb-girdle muscular dystrophy), 8 with inflammatory idiopathic myopathies (idiopathic body myositis, dermatomyositis), and 2 with congenital myopathies (central core disease). One fall occurred in one participant while wearing the device during the familiarization period on the third visit before evaluation started, without injury. Three participants did not pursue after the first visit: thigh pain unrelated to the first visit (n = 1), timetable issue (n = 2). Two participants did not perform the third visit due to scheduling conflicts. Predicted muscle strength values for the lower limbs are displayed in Fig. 1. MVC_pred_ was 27.6 ± 42.9% for knee extension, 35.0 ± 59.3% for knee flexion, 85.3 ± 53.5% for hip extension, 47.9 ± 47.6% for hip flexion, 126.4 ± 17.6% for ankle plantar flexion, and 3.9 ± 8.3% for ankle dorsal flexion. 2MWT was 70.6 ± 22.4% of predicted distance.

### Severity of lower limb muscle weakness and effect on the Keeogo on performance

Performances in 2MWT, STS30, 10mWT, and SWAY are displayed in Fig. 1. The 2MWT distance and predicted distance was significantly lower with large effect size (Cohen’s d = 1.58 and 1.52, respectively) with the Keeogo (106.5 ± 51.3 vs. 134.0 ± 31.0 m; 56.1 ± 28.2 vs. 70.6 ± 22.4% of predicted value, both p < 0.0001). 10mWT when using the Keeogo was significantly increased (11.5 ± 4.3 vs. 9.0 ± 2.1 s, p < 0.001) with large effect size (Cohen’s d = -0.92). During the 2MWT and 10mWT, gait speed, stride length and cadence were significantly decreased when using the Keeogo with large effects (all p < 0.001, Cohen’s d in Table 1). Step duration was significantly increased with large effect size (p < 0.001, Cohen’s d = −1.03). No statistical difference was found regarding asymmetries of gait cycle duration, step duration, and stride length (all p > 0.21, Table 1).

**Table 1:** Spatio-temporal gait parameters during the 2-min walk test and the 10-m walk test without and with the Keeogo

	Tests	Without	With	p	Magnitude
Cadence (steps/min)	2MWT	111.8 ± 22.9	93.4 ± 23.5	***	Large
	10mWT	111.4 ± 19.2	90.6 ± 18.9	***	Large
Gait cycle duration (s)	2MWT	1.1 ± 0.3	1.3 ± 0.3	***	Large
	10mWT	1.1 ± 0.2	1.3 ± 0.3	***	Large
Gait speed (m/s)	2MWT	1.1 ± 0.3	0.9 ± 0.5	***	Large
	10mWT	1.1 ± 0.3	0.9 ± 0.4	***	Large
Gait cycle duration asymmetry	2MWT	0.0 ± 0.0	0.0 ± 0.1	ns	Small
	10mWT	0 .0 ± 0.2	0.0 ± 0.2	ns	Negligible
Step duration (s)	2MWT	0.5 ± 0.1	0.6 ± 0.2	***	Large
	10mWT	0.5 ± 0.1	0.7 ± 0.1	**	Large
Step duration asymmetry	2MWT	0.5 ± 0.8	0.9 ± 0.7	ns	Small
	10mWT	0.7 ± 0.8	1.4 ± 1.4	ns	Small
Stride length (m)	2MWT	1.3 ± 0.2	1.1 ± 0.2	****	Large
	10mWT	1.3 ± 0.2	1.2 ± 0.3	***	Large
Stride length asymmetry	2MWT	0.4 ± 0.8	0.5 ± 0.5	ns	Small
	10mWT	0.7 ± 0.8	0.9 ± 0.7	ns	Moderate

Data are shown as median ± IQR. *ns* not significant; **, p < 0.01; ***, p < 0.001; ****, p < 0.0001; *ns* not significant, *d* effect size as computed with Cohen’s d. 2MWT, 2-min walk test (n = 16), 10mWT, 10-min walk test

There was no statistical difference between conditions during STS30 (7 ± 3 vs. 8 ± 4 repetitions, p < 0.07). No statistical difference was found between conditions in postural sway variables (Table 2, all p > 0.37).

**Table 2:** Spatio-temporal parameters during static postural stability without and with the Keeogo

	Without	With	p	*d*	Magnitude
95% ellipse axis 1 (m.s^−2^)	0.04 ± 0.02	0.04 ± 0.05	ns	− 0.11	Negligible
95% ellipse axis 2 (m.s^−2^)	0.12 ± 0.05	0.13 ± 0.04	ns	0.05	Negligible
Mean velocity (m.s^−1^)	0.10 ± 0.06	0.10 ± 0.08	ns	0.19	Negligible
rms sway (m.s^−2^)	0.06 ± 0.02	0.06 ± 0.01	ns	0.06	Negligible
Sway area (m^2^.s^−4^)	0.02 ± 0.01	0.02 ± 0.02	ns	-0.18	Negligible

Data are shown as median ± IQR; *ns* not significant, *d* effect size as computed with Cohen’s d with 0.2 = small effect. 0.5 = moderate effect. 0.8 = large effect.

### Changes in perceived stability, exertion, dyspnea, and pain

**Fig. 2:**
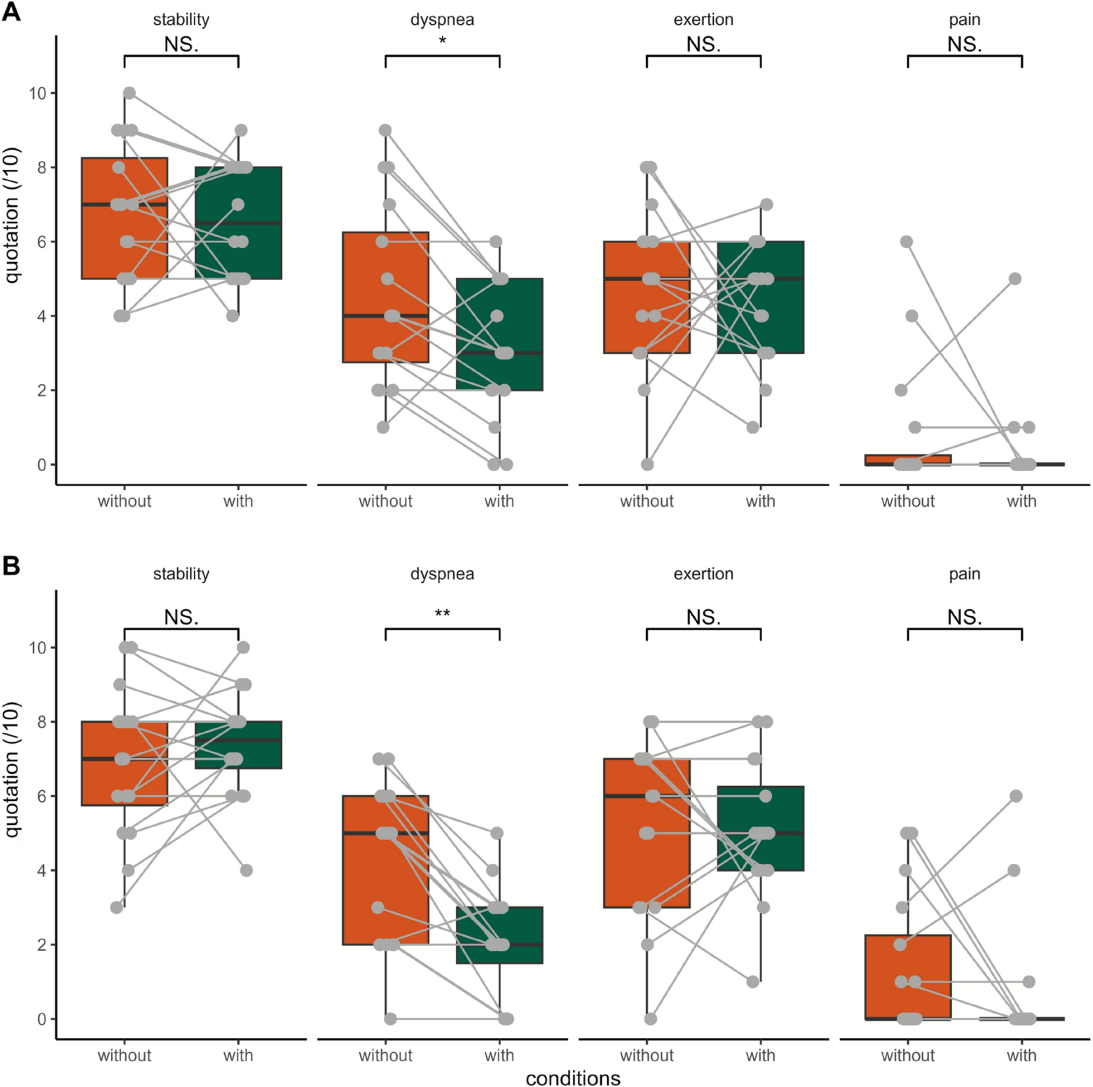
Comparison of verbal analog scale quotes of stability, exertion, pain, and dyspnea without and with the Keeogo during 2-min walk test (**A**) and 30-s sit-to-stand (**B**). Quotes are displayed as boxes and whiskers plots, with the median (thick middle line), interquartile range (end of boxes) and vertical as range between maximal and minimal value Each participant is represented by a point in both the with and without exoskeleton conditions, connected by a line. *, p < 0.05. n = 16

**Fig. 3:**
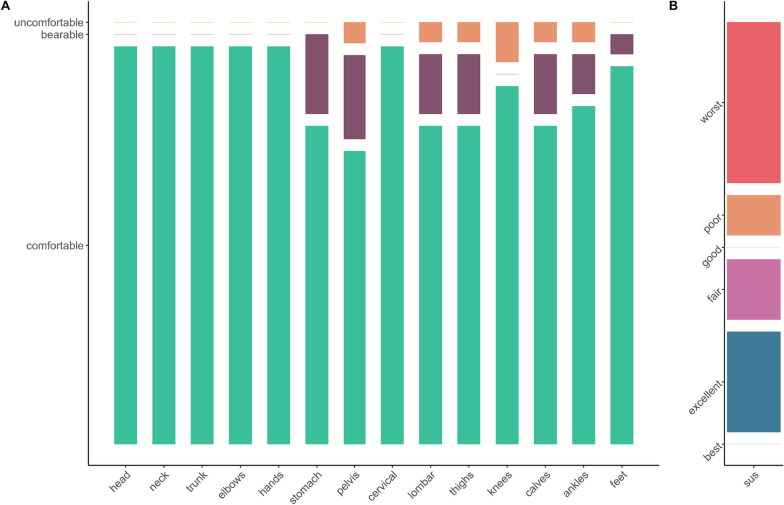
Perceived comfort when using the Keeogo as assessed with the modified Nordic questionnaire (**A**) and the usability of the device assessed with the system usability scale (**B**). In **A**, the scale is divided in 3 categories for each body part: comfortable (green), bearable (purple) and uncomfortable (orange). In **B**, the system usability scale (SUS) is divided in 6 categories of usability: best (green), excellent (dark blue), fair (pink), good (light blue), poor (orange) and worst imaginable (red). The range reflects the number of participants falling into each category

Perceptions with and without the Keeogo at the end of the STS30 and 2MWT are shown in Fig. 2. Perceived dyspnea when using the Keeogo during STS30 and 2MWT was significantly lowered with moderate effect size (2 ± 2 vs. 5 ± 4 and 3 ± 3 vs. 4 ± 4, respectively, both p < 0.05). No statistical difference in perceived stability, exertion, and pain was found for other tests (all p > 0.08). Results for the SUS and NMQ are displayed in Fig. 3. Comfort questionnaires indicated that a minority of participants reported discomfort while wearing the device, and half rated its usability as “poor” to “worst” (n = 10/18).

### Joint kinematics during gait and sit-to-stand/stand-to-sit transfers

**Fig. 4:**
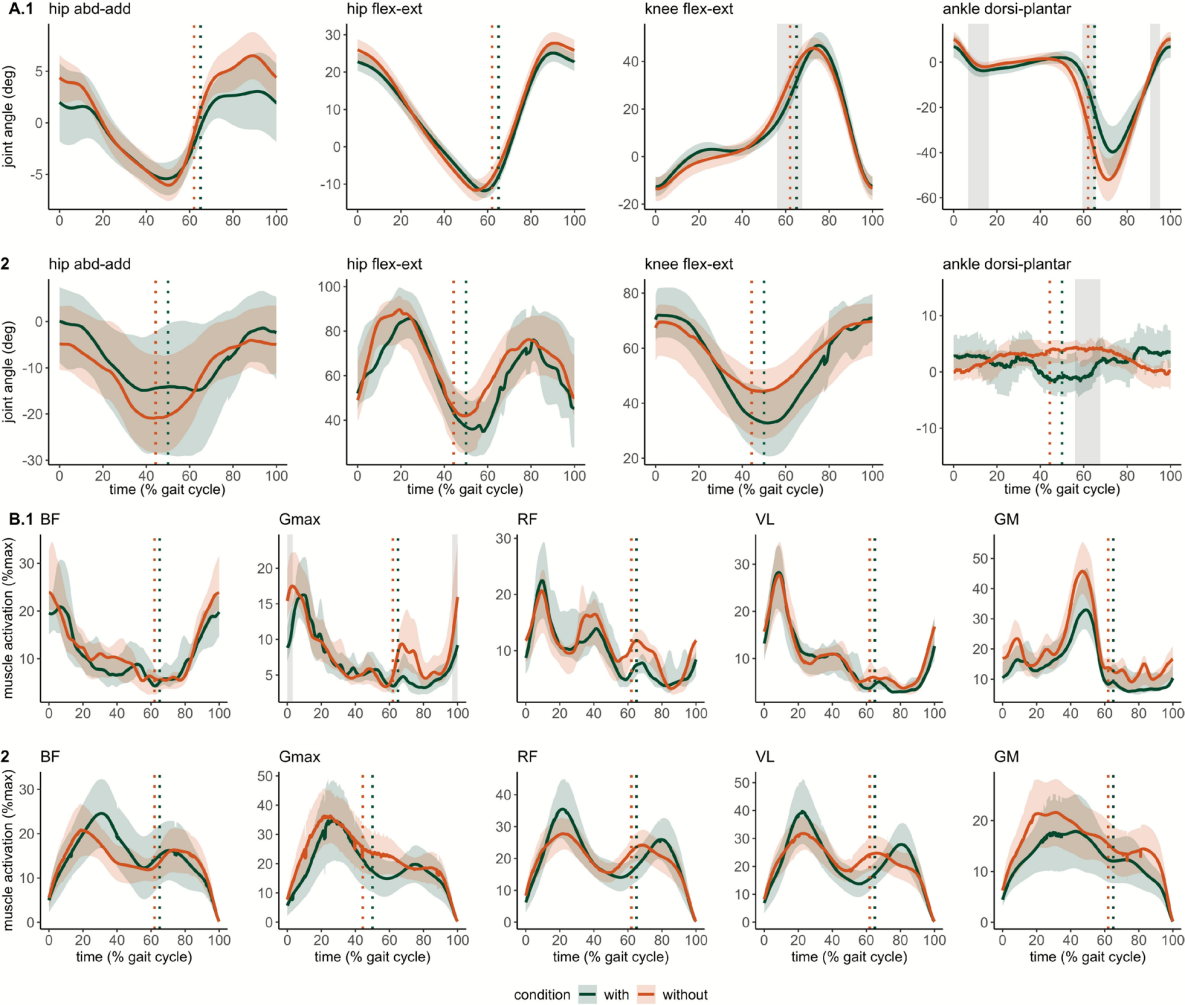
Average time-varying (all participants) joint angle and electromyography (EMG) patterns measured during gait cycle (**A** and **B**, respectively) during the 2-min walk test (2MWT, A.1, B.1) and 30-s sit-to-stand (STS30, A.2, B.2). **A** represents joint angle amplitude data for hip adduction-abduction, hip and knee flexion–extension and ankle dorsi- and plantar-flexion. **B** displays EMG amplitude for biceps femoris (BF), gluteus maximus (Gmax), rectus femoris (RF), vastus lateralis (VL) and gastrocnemius medialis (GM). Thick lines are average joint angle and average normalized EMG (**A** and **B**, respectively) and shaded areas are the corresponding confidence intervals (95%). At 0% on the x-axis occurred heel strike in 2MWT (A.1 and B.2) and start of sit-to-stand in STS30 (B.1 and B.2). Vertical dashed lines show the average time of toe-off during the gait cycle and the sit-to-stand and stand-to-sit transition in STS30. Based on statistical parametric mapping results, grey areas show the periods when a significant difference was observed between conditions without and with the use of the Keeogo in joint range of motion and EMG amplitude. All p < 0.05. *abd* abduction, *add* adduction, *flex* flexion, *ext* extension, *dorsi* dorsiflexion, *plant* plantarflexion, *deg* degree

JA during 2MWT was not available for two participants and for one participant during STS30 due to technical issues. Joint angles during gait cycle during the 2MWT and STS30 are displayed in Fig. 4. Range of motion for each measured joint and movement during both 2MWT and STS30 tests are displayed in Table 3.

**Table 3:** Range of motion for hip, knee, and ankle without and the Keeogo in stance and swing phases during the gait cycles of the 2-min walk test and in sit-to-stand and stand-to-sit phases during the 30-s sit-to-stand test

	Tests	Phase	Without	With	*p*	*d*
Hip abduction/adduction (degree)	2MWT	Stance	25.8 ± 16.9	22.1 ± 9.8	ns	Negligible
		Swing	12.7 ± 6.9	10.4 ± 8.0	ns	Negligible
	STS30	Sit-to-stand	51.9 ± 37.0	44.3 ± 37.2	ns	Small
		Stand-to-sit	59.1 ± 33.8	45.4 ± 32.9	ns	Small
Hip flexion/extension (degree)	2MWT	Stance	50.6 ± 13.1	47.0 ± 9.2	ns	Negligible
		Swing	36.9 ± 10.5	32.7 ± 7.6	*	Moderate
	STS30	Sit-to-stand	77.7 ± 27.9	80.7 ± 24.1	ns	Negligible
		Stand-to-sit	78.9 ± 25.6	76.4 ± 26.4	ns	Small
Knee flexion/extension (degree)	2MWT	Stance	79.8 ± 13.0	90.4 ± 17.1	ns	Small
		Swing	67.9 ± 9.9	69.6 ± 10.2	ns	Negligible
	STS30	Sit-to-stand	35.3 ± 25.5	47.6 ± 26.1	ns	Negligible
		Stand-to-sit	38.6 ± 23.5	46.9 ± 27.0	ns	Negligible
Ankle flexion/extension (degree)	2MWT	Stance	108.0 ± 35.5	95.7 ± 55.7	ns	Negligible
		Swing	78.3 ± 56.0	67.9 ± 41.4	ns	Small
	STS30	Sit-to-stand	14.0 ± 6.3	13.0 ± 7.0	ns	Negligible
		Stand-to-sit	14.7 ± 6.2	13.1 ± 7.2	ns	Small

Data are shown as median ± IQR. *, p < 0.05; *ns* not significant; *d* effect size as computed with Cohen’s d with 0.2 = small effect. 0.5 = moderate effect. 0.8 = large effect. 2MWT, 2-min walk test, STS30, 30-s sit-to-stand.

**Fig. 5:**
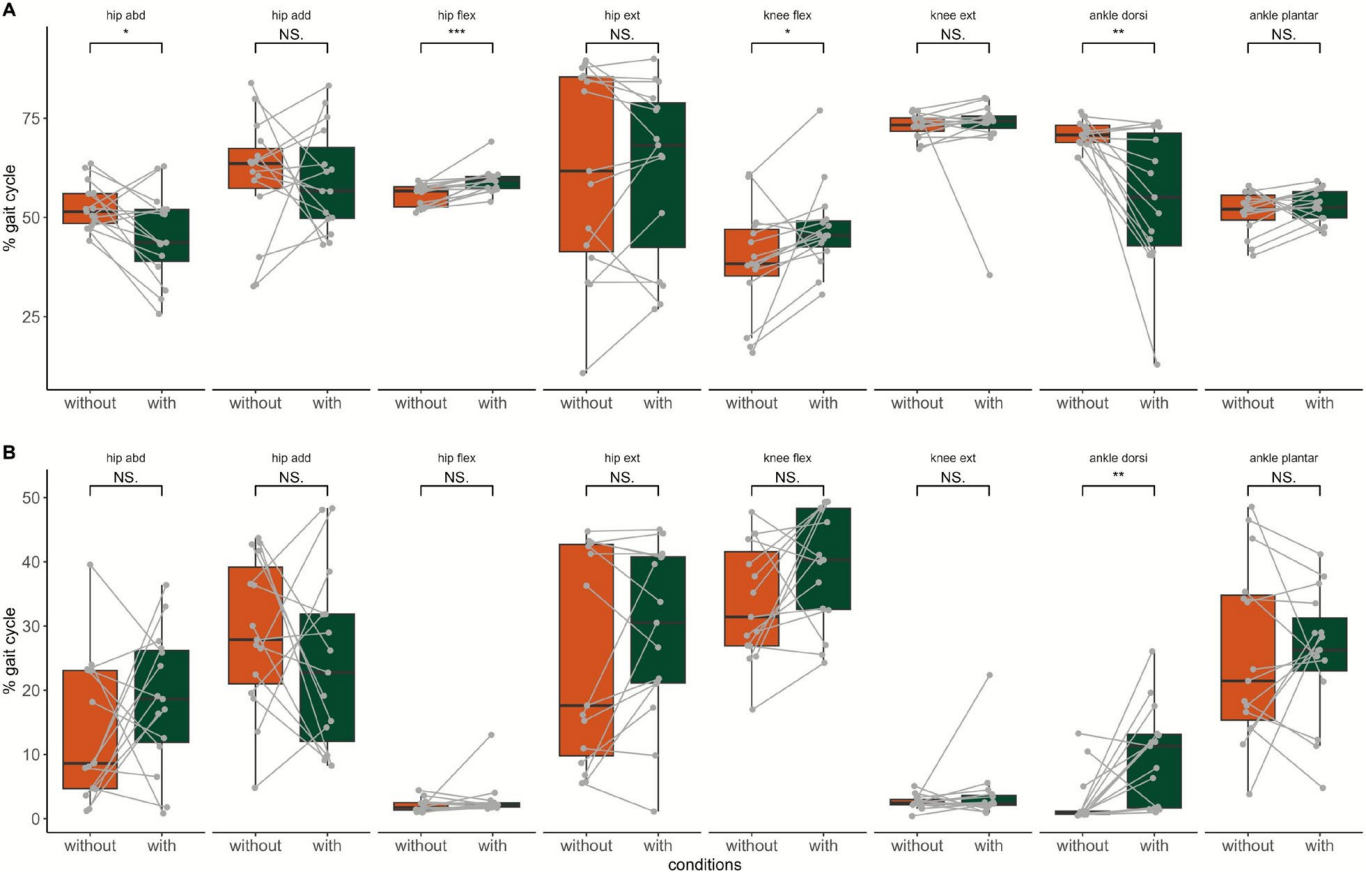
Comparison of average time (**A**) and standard deviation’s (**B**) of peak occurrence (in percentage of gait cycle) of the hip abduction, hip adduction, hip flexion, hip extension, knee flexion, knee extension, ankle dorsiflexion and plantarflexion. The average time peak occurrence is the time when the joint motion is at its peak angle. Box and whisker plots, without in red and represent the distribution of the values, thick line is the median, thin lines of the box are interquartile, and vertical line is the range between maximal and minimal value Each participant is represented by a point in both the with and without exoskeleton conditions, connected by a line. flex, flexion; plantar, plantarflexion; abd, abduction; ext, extension; dorsi, dorsiflexion *, p < 0.05; **, p < 0.01 and ***, p < 0.001; *NS* not significant. n = 16

Hip flexion-extension_RoM_ was significantly lowered with moderate effect size when using the Keeogo during the swing phase. Regarding STS30, hip flexion-extension_RoM_ was significantly decreased with the Keeogo during sit-to-stand and stand-to-sit transitions with small effect size. No other statistical difference in range of motion was found. Results of statistical parametric mapping for joint angles are presented in Fig. 4. A significant condition × time (percentage of the gait cycle) interaction was found for ankle dorsi- and plantarflexion (all p < 0.05) with three blocks where ankle differ between condition without and with the use of the Keeogo. JA_avg_ and JA_SD_ peak time during gait cycle are shown in Fig. 5. A significantly larger ankle-dorsiflexion_SD_ was found when using the Keeogo (11.33 ± 11.42% vs 0.98 ± 0.63% of gait cycle, p < 0.01). A significant delay was found for knee-flexion_avg_, hip-flexion_avg_, hip-extension_avg_ peak with moderate to large effect size (all p < 0.05). Also, ankle-dorsiflexion_avg_ and hip-abduction_avg_ occurred significantly sooner when using the Keeogo with moderate to large effect size (both p < 0.05). No statistical difference was found for any of the asymmetry parameters. Spatiotemporal gait parameters during 2MWT and 10mWT are presented in Table 4. Percent of the gait cycle for double support, single limb support, swing and stance were significantly increased during both 2MWT and 10mWT (all p < 0.001). During STS30 the time duration of sit-to-stand phase was significantly longer when using the Keeogo (53.4 ± 6.2% vs 42.0 ± 10.4% of sit-to-stand-to-sit cycle, p < 0.05).

**Table 4:** Comparison of gait parameters of performance without and with the Keeogo during 2-min walk test and 10-m walk test

	Tests	Without	With	*p*	*d*
Circumduction (cm)	2MWT	3.1 ± 2.4	3.6 ± 1.7	ns	Small
	10mWT	3.7 ± 3.1	3.5 ± 2.0	ns	Negligible
Double support (%)	2MWT	22.8 ± 6.4	29.3 ± 9.1	****	Large
	10mWT	24.0 ± 8.4	32.2 ± 8.6	***	Large
Elevation at mid-swing (cm)	2MWT	2.6 ± 1.9	2.6 ± 2.4	ns	Negligible
	10mWT	3.3 ± 2.1	3.1 ± 2.3	ns	Small
Foot strike angle (degrees)	2MWT	20.8 ± 6.2	18.8 ± 10.0	*	Moderate
	10mWT	21.4 ± 7.2	19.6 ± 9.2	**	Moderate
Lateral step variability (cm)	2MWT	4.5 ± 1.1	4.4 ± 0.8	ns	Negligible
	10mWT	4.1 ± 1.7	3.9 ± 0.6	ns	Negligible
Single limb support (%)	2MWT	38.6 ± 3.2	35.4 ± 3.5	***	Large
	10mWT	37.8 ± 4.2	33.8 ± 4.5	***	Large
Stance (%)	2MWT	61.4 ± 3.2	64.7 ± 3.4	***	Large
	10mWT	61.9 ± 4.2	66.1 ± 4.2	***	Large
Stance asymmetry	2MWT	0.3 ± 0.4	0.3 ± 0.6	ns	Negligible
	10mWT	0.3 ± 0.4	0.4 ± 0.5	ns	Negligible
Swing (%)	2MWT	38.6 ± 3.2	35.3 ± 3.4	***	Large
	10mWT	38.1 ± 4.2	34.0 ± 4.2	***	Large
Swing asymmetry	2MWT	0.6 ± 0.6	0.6 ± 1.2	ns	Small
	10mWT	0.5 ± 0.6	0.7 ± 1.0	ns	Negligible
Terminal double support (%)	2MWT	11.4 ± 3.2	14.7 ± 4.6	****	Large
	10mWT	12.0 ± 4.2	16.0 ± 4.4	**	Large
Toe off angle (degrees)	2MWT	38.6 ± 5.0	32.5 ± 9.8	***	Large
	10mWT	38.6 ± 4.2	30.7 ± 6.0	**	Large
Toe out angle (degrees)	2MWT	7.2 ± 9.1	9.4 ± 10.8	*	Moderate
	10mWT	5.8 ± 10.1	8.3 ± 13.3	*	Moderate
Toe off timing (%)	2MWT	61.8 ± 2.9	65.2 ± 5.4	***	Large
	10mWT	61.6 ± 3.7	64.8 ± 3.8	***	Large

Data are shown as median ± IQR. *%*, percentage, *cm* centimeter. *, p < 0.05; **, p < 0.01; ***, p < 0.001; ****, p < 0.0001; *ns* not significant, *d* effect size as computed with Cohen’s d with 0.2 = small effect. 0.5 = moderate effect. 0.8 = large effect. 2MWT, 2-min walk test (n = 16), 10mWT, 10-min walk test.

### Muscle activation patterns during gait and sit-to-stand/stand-to-sit transfers

**Fig. 6:**
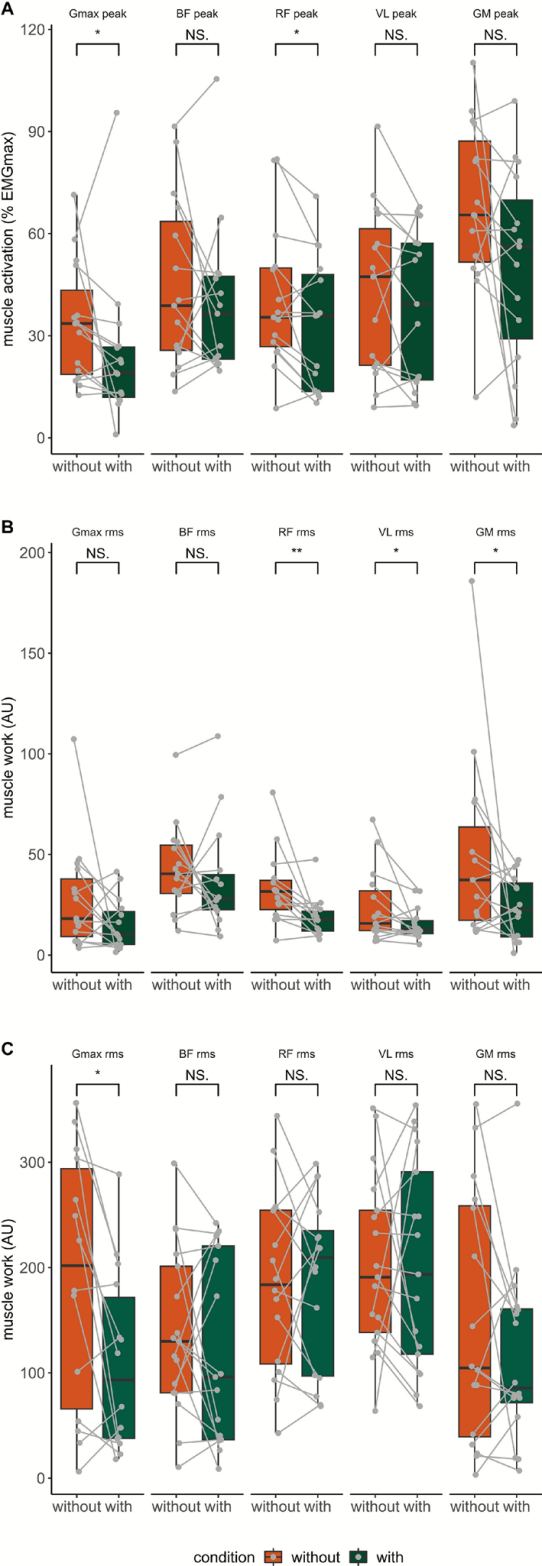
Comparison of maximal muscle activation overall gait cycle (**A**) and muscle work during swing phase (**B**) peak occurrence (in percentage of gait cycle) and muscle work overall 30-s sit-to-stand test (**C**) of the Gluteus Maximus (Gmax), Biceps Femoris (BF), Rectus Femoris (RF), Vastus Lateralis (VL) and Gastrocnemius Medialis (GM). Box and whisker plots represent the distribution of the peak normalized muscle activation, thick line is the median, thin lines of the box are interquartile, and vertical line is the range between maximal and minimal value. Each participant is represented by a point and a line linked condition without and with the use of the Keeogo. *, p < 0.05; **, p < 0.01; *NS* not significant; *AU* arbitrary unit

EMG during 2MWT were not available for two participants and for one participant during STS30 due to technical issues. Peak activation and muscle workload are presented in Table 5, Fig. 4, and Fig. 6. Gmax_peak_ was significantly reduced when wearing the device during both stance and swing phases with moderate effect size (p < 0.05 and p < 0.01, respectively). RF_peak_ was significantly increased when wearing the exoskeleton during both stance and swing phases with moderate effect size (p < 0.05 and p < 0.01, respectively). During the swing phase VL_peak_ was reduced while wearing the exoskeleton with moderate effect size (p < 0.05). GM_AUC_, VL_AUC_, RF_AUC_, were decreased during swing phase with the Keeogo with small to moderate effect size (all were p < 0.05). Higher RF_AUC_ overall gait cycle was found while using the exoskeleton with moderate effect size (p < 0.05). No statistical differences were found for BF_AUC_ and Gmax_AUC_. Regarding, EMG_avg_ and EMG_SD_, no statistical differences were found for any muscle during the 2MWT (all p > 0.1). During the STS30, the Gmax_AUC_ was significantly reduced with the device with moderate effect (176.5 ± 195.8 vs 231.6 ± 172.6 mV.s, p < 0.05). No other statistical difference was found in EMG_peak_ and EMG_AUC_ during STS30. No statistical differences in median power frequency were observed in any studied muscle (all p > 0.1). Statistical parametric mapping of joint angle is shown in Fig. 4. A significant condition x time interaction in Gmax muscle was found.

**Table 5:** Normalized electromyographic activity of measured muscles in conditions without and with the Keeogo during 2-min walk test

		Phase	Without	With	*p*	*d*
Gmax	Peak (%)	Stance	27.6 ± 17.5	19.1 ± 15.2	*	Small
		Swing	19.0 ± 23.0	7.7 ± 10.0	**	Moderate
	Load (AU)	Stance	50.3 ± 31.1	42.9 ± 43.6	ns	Negligible
		Swing	17.4 ± 25.0	10.1 ± 16.4	ns	Small
BF	Peak (%)	Stance	29.8 ± 38.0	33.2 ± 17.7	ns	Small
		Swing	33.8 ± 23.1	20.1 ± 17.1	ns	Small
	Load (AU)	Stance	49.5 ± 66.5	48.5 ± 33.8	ns	Small
		Swing	41.7 ± 27.5	27.9 ± 17.4	ns	Small
RF	Peak (%)	Stance	34.7 ± 18.5	35.8 ± 35.3	*	Moderate
		Swing	21.2 ± 11.1	13.3 ± 8.2	**	Large
	Load (AU)	Stance	63.2 ± 65.6	91.6 ± 72.0	ns	Small
		Swing	27.1 ± 16.7	17.8 ± 9.9	**	Moderate
VL	Peak (%)	Stance	30.0 ± 36.9	39.3 ± 40.3	ns	Small
		Swing	15.7 ± 14.5	11.4 ± 8.0	*	Moderate
	Load (AU)	Stance	68.5 ± 74.1	68.6 ± 79.7	ns	Negligible
		Swing	15.1 ± 19.4	12.9 ± 6.5	*	Moderate
GM	Peak (%)	Stance	64.3 ± 36.9	55.8 ± 40.8	ns	Small
		Swing	25.6 ± 23.2	13.0 ± 18.6	ns	Small
	Load (AU)	Stance	49.5 ± 66.5	48.5 ± 33.8	ns	Small
		Swing	41.7 ± 27.5	27.9 ± 17.4	ns	Small

Data are shown as median ± IQR. *Gmax* Gluteus Maximus, *BF* Biceps Femoris, *RF* Rectus Femoris, *VL* Vastus Lateralis, *GM* Gastrocnemius Medialis, *%* percent of maximum electromyography signal, *AUC* area under the curve, *AU* arbitrary unit. *ns* non-significant: **, p < 0.01; ***, p < 0.001; ****, p < 0.0001. d, effect size as computed with Cohen’s d with 0.2 = small effect. 0.5 = moderate effect. 0.8 = large effect

### Determinants of impaired performance when using the Keeogo during the 2MWT and STS30

**Fig. 7:**
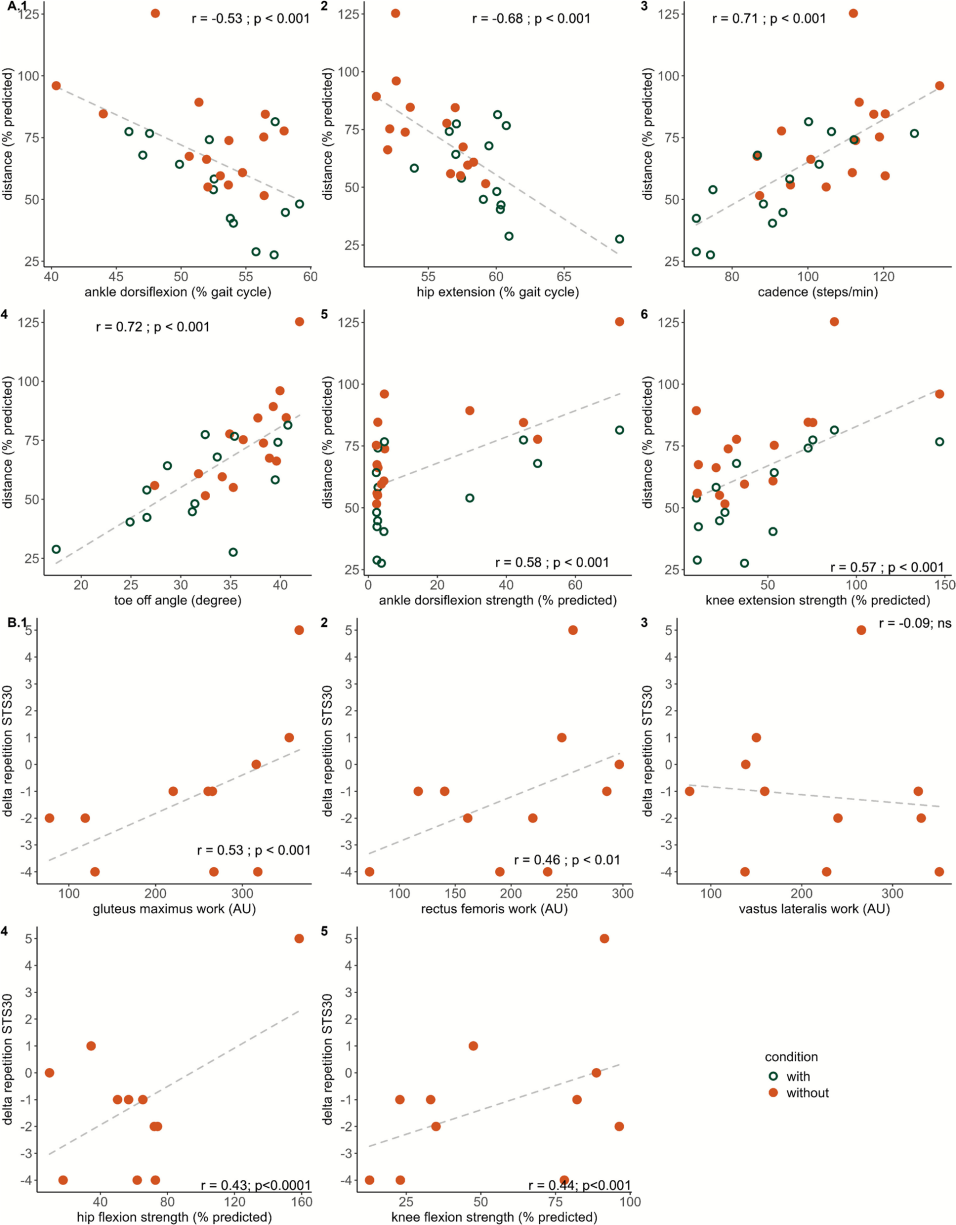
Relationship between (**A**) 2-minutes walking distance (2MWT) and most selected features in penalized least absolute shrinkage and selection operator (LASSO) regression model and (**B**) in 30-second sit-to-stand (STS30) repetitions and selected features in penalized LASSO regression model in participants with neuromuscular diseases when using or not the Keeogo. The 2MWT distance in relationship with average ankle plantarflexion peak occurrence (A.1), average hip flexion peak occurrence (A.2), cadence (A.3), toe-off angle (A.4), ankle dorsiflexion predicted strength (A.5), and knee extension predicted strength (A.6). The delta repetition in STS30 in relationship with gluteus maximus, rectus femoris and vastus lateralis muscle activation work (B.1-3), and hip and knee flexion strength (B.4 -5). Each participant is represented by an orange point (without Keeogo), and a green circle (with Keeogo) during 2MWT (A.1-6) and each participant is represented by an orange point (delta performance) in STS30 (B.1-5). Correlation coefficient (r) and p values are shown

The results presented in this section for the 2MWT were processed by including data without and with the Keeogo. Significant positive relationships between the 2MWT_pred_ the device and predicted muscle strength in knee flexors/ extensors and ankle flexors/extensors were found (all r > 0.32 and p < 0.01; Fig. 7). Gait parameters were associated with the magnitude of decreased gait performance when using the device (Fig. 7A3, 4). Lower cadence (r = 0.71, p < 0.001), lower gait speed (r = 0. 88, p < 0.001), and shorter stride length (r = 0.73, p < 0.001) were related to smaller 2MWT_pred_ without using the device. Toe off time was negatively associated to 2MWT_pred_ without the device (r = −0.71, p < 0.001). Ankle angle at toe off and ankle-dorsiflexion_avg_ were positively correlated with greater 2MWT_pred_ without the device (r = 0.72, p < 0.01 and 0.52, p < 0.01, respectively) and ankle-plantarflexion_avg_ was negatively correlated with 2MWT_pred_ (r = −0.53, p < 0.01).

**Fig. 8:**
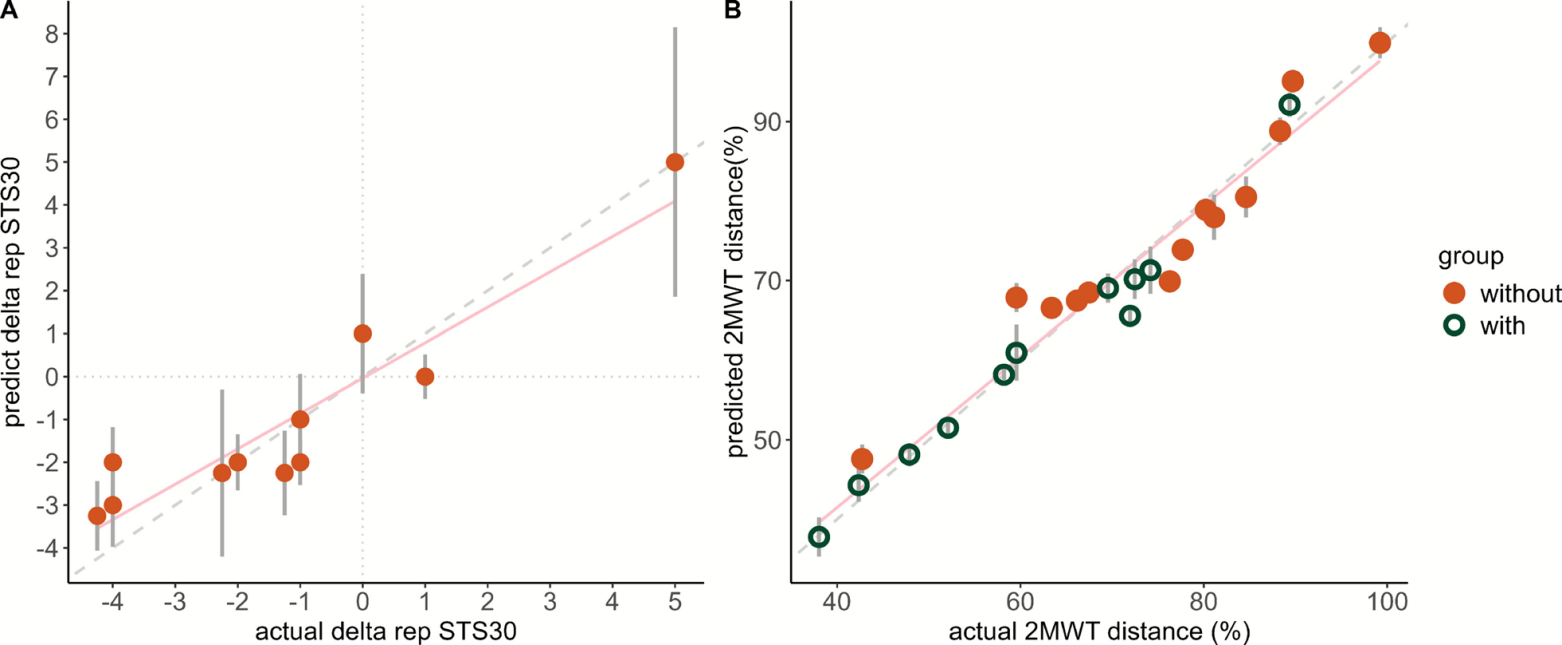
Penalized least absolute shrinkage and selection operator (LASSO) regression prediction to predict 2-minute walk test distance (**B**) and during 30-second sit-to-stand (**A**). A represents the comparison between actual and predicted percentage of 2MWT predicted distance. Circle (without) and diamond (with) represented participants, and a unique color is attributed to each participant (n= 29). **B** represents the comparison between actual and predicted delta repetitions with versus without the Keeogo during STS30. Circle represented participants (n= 11). Vertical grey associated is the variance of their own prediction, dashed grey line corresponds to the identity line, and pink line is line of the penalized LASSO regression. Model predicting 2MWT distance used the step duration, toe-off angle, perceived stability, ankle dorsiflexion and knee extension strength and average in ankle dorsiflexion, plantarflexion, and hip flexion peak occurrence and model predicting delta STS30 used hip and knee flexion strength and Rectus Femoris and Vastus Lateralis activation work

Penalized LASSO regression model results are displayed in Fig. 8 and Table 6 for both 2MWT_pred_ and delta STS30. In the first 1000 LASSO models, predicted ankle dorsiflexion and knee extension strength, cadence, hip flexion_avg_, ankle plantarflexion_avg_, and ankle angle at toe-off were selected over seventy percent of the time from the best half LASSO models for 2MWT. The performances of the models with all features versus 6 features were compared using R^2^ and reduced overfitting was observed (R^2^ = 0.74 with all features and R^2^ = 0.84 with 6). The performances of the 500 best models were as follows: MSE was 49.7 ± 29.6, R^2^ was 0.84 ± 0.14 and RMSE 6.7 ± 2.1% 2MWT_pred_ and MAE was 5.5 ± 1.7% 2MWT_pred_. Variances for each participant between actual and predicted 2MWT_pred_ and actual versus predicted delta performance STS30_pred_ from both models is displayed in Fig. 8. The performance of the final LASSO model with the entire dataset was as follows: MSE = 24.5, R^2^ = 0.94, and MAE = 3.9% 2MWT_pred_. Regarding change in STS30, the first 1000 LASSO models selected predicted strength in hip and knee flexion, RF_AUC_, Gmax_AUC_, and VL_AUC_. The performance between the model using 5 features versus the model using all features of the 500 best models were as follows: R^2^ was 0.54 ± 0.25 vs. 0.35 ± 0.22, MSE was 4.9 ± 4.0 vs. 9.0 ± 6.8, RMSE was 2.08 ± 0.77 vs 2.77 ± 1.13 delta STS30_pred,_ and MAE was 1.79 ± 0.71 vs. 2.56 ± 1.03% delta STS30_pred_. The performance of the final LASSO model using the entire dataset was as follows: MSE = 0.78, R^2^ = 0.88, and MAE was 0.75 delta STS30_pred_.

**Table 6:** Penalized least absolute shrinkage and selection operator (LASSO) regression equation parameters predicting change in 2-min walk test and 30-s sit-to-stand with the Keeogo^+^

		β	n	r	p
2MWT	(Intercept)	164.7700	–	–	–
	Ankle plantarflexion_avg_ (% gait cycle)	−0.4790	29	−0.53	****
	Hip flexion_avg_ (% gait cycle)	−2.2320	29	−0.68	****
	Cadence (steps/min)	0.1723	29	0.71	****
	Toe off angle (degree)	0.7397	29	0.72	****
	Ankle dorsiflexion strength (%pred)	0.3833	29	0.58	****
	Knee extension strength (%pred)	0.1133	29	0.57	****
STS30	(Intercept)	−7.7186	–	–	–
	Hip flexion strength (%pred)	0.0421	11	0.53	****
	Knee flexion strength (%pred)	0.0226	11	0.44	*
	RF_AUC_ (arbitrary unit)	0.0199	11	0.45	**
	VL_AUC_ (arbitrary unit)	−0.0112	11	−0.1	ns
	Gmax_AUC_ (arbitrary unit)	0.0050	11	0.53	***

*%* percent, *min* minute; *pred* predicted value, *β* Beta, *R* coefficient of correlation, *ns* non-significant. *, p < 0.05; **, p < 0.01; ****, p < 0.0001*

## Discussion

This study aimed to investigate the effects of the knee-powered lower limbs exoskeleton Keeogo on gait, sit-to-stand/stand-to-sit transitions, and static postural stability in adults with NMD. To the best of our knowledge, this is the first study to comprehensively assess Keeogo efficacy perceptions, gait parameters, lower limb kinematics, and muscle activation in gait, sit-to-stand transitions and static stability within a group of individuals with NMD. As hypothesized, significant decreases in performance were found during gait and sit-to-stand transition at the group level. Only a minority of participants equaled or improved their performance during 10mWT and STS30 (Fig. 1). The following variables were identified as determinants of impaired 2MWT performance: smaller gait speed, stride length, and cadence, prolonged step duration, and lower toe-off angle. Additionally, the study also hypothesized that participants with less severe impairments would experience greater beneficial effects. Our findings support this as the severity of knee extensors and ankle dorsiflexors weakness were identified as critical factors of decreased walking performance when using the device. During the STS30, smaller hip flexion and knee flexion strength were associated with the magnitude of performance impairments with the Keeogo. Smaller RF and Gmax muscle activation without device were also identified as determinants of impaired STS30 when using the Keeogo.

### The use of Keeogo did not affect static postural stability

Our results showed no significant differences during quiet stance with the Keeogo. These results were observed for all main variables (i.e. the center of mass velocity, area, and acceleration). Therefore, the powered assistance at the knee level does not seem to affect static postural stability. In the present work, the postural SWAY was tested with eyes open, reducing the odds to detect significant changes in COP velocity due to visual compensation [30]. Accordingly, the perception of stability was unchanged, suggesting that the use of this device does not impair safety, although it does not appear to improve it either. One must note that the perceived stability without the Keeogo was high so that the detection of potential improvement may have been limited by the ceiling effect of verbal scales. Heightened activation of lower limb muscles, particularly the GM muscle, has been reported in healthy individuals when weight was added in a static position [31]. This discrepancy might be due to the compensation of the device’s weight by the ongoing assistance provided to the knees. Finally, the level of muscle activation in both conditions was relatively low, which may have hindered the detection of differences.

### Effects of Keeogo on gait and sit-to-stand/stand-to-sit transitions

As hypothesized, gait performance during the 2MWT was reduced when using the device. The reduced performances with assistance are in line with the previous work of McGibbons et al. in participants with multiple sclerosis that showed small decrement in 6MWT when using the keeogo [7]. Despite familiarization sessions, participants were still relatively inexperienced with using the device. This inexperience likely contributed to the observed performance decrements. McGibbons et al. noted progression in unassisted performance over time, attributing this to both the learning curve associated with using the device and its rehabilitative effects [7]. Therefore, it cannot be ruled out that extended use and increased familiarity with the Keeogo could potentially lead to enhanced gait performance in the studied participants. However, the mass, inertia and inherent stiffness of the device previously documented in healthy participants also contribute to explain performance impairments in this group with substantial muscle weakness [32]. During the 2MWT, the mean decrease in distance was –34 m, which was greater than the decrease observed by McGibbon et al. (i.e., −23 m during a 6MWT) [7]. Increase in gait performance with the Keeogo has been reported in another study in participants with neurological impairments (i.e. multiple sclerosis, spinal cord injury, stroke, cerebellar ataxia, cerebral palsy) but only in those with moderate functional impairments [8]. Our findings may be partly explained by the fact that individuals exhibited more severe and more largely distributed muscle impairments. As described in previous studies, stride length was lower when using the device and was responsible for lower gait speed [7]. The reduction in stride length was not compensated for by an increase in cadence. Unlike self-supporting or multi-joint powered exoskeletons that have demonstrated improvements in walking speed and gait symmetry in other populations [4, 5], the Keeogo provides powered assistance only at the knee and requires users to bear its full weight. These design characteristics, combined with the diffuse muscle weakness typical of NMD, likely to explain the absence of performance improvement in our participants. In contrast, McGibbon et al. [32] observed preserved gait velocity and reduced knee adduction moments in healthy adults using the same device, supporting the notion that sufficient baseline strength is required to benefit from its assistance [32]. The absence of a healthy control group in the present study limits direct comparisons, but our findings underscore that device design and population-specific impairments are key determinants of functional outcomes.

In contrast with previous works, we assessed changes in movement patterns and muscle activation during gait and chair rising/sitting when using the Keeogo. Interestingly, reduced muscle activation was observed in knee extensors (RF and VL) during the swing phase suggesting that the assistance provided to the knee during the swing phase in knee extension successfully alleviated knee extensors. Surprisingly, RF muscle activation was increased with the device during the stance phase. Similar findings in post-stroke participants with another device were previously reported [33]. The increased RF peak activity was found to occur at the beginning of the stance phase, just after heel strike suggesting that added weight may increase the knee extensors muscle load to avoid knee buckling (Fig. 4). This result contrasts with previous assertions which indicated the possibility of a reduced knee muscle activation between synergists [32]. The inability of the participants to fully exploit the aid provided by the device may also explain this counter-intuitive finding. No difference was found for the BF whereas a reduction in the hip extensor Gmax was found as well as reduced knee flexor GM muscle. The device assistance likely favored the reduction of Gmax muscle activation over equally reducing hip extensors which may explain the absence of changes in BF muscle activation. Previous study in healthy subjects observed that BF was activated mostly at the end of the swing phase, while Gmax was at the beginning of the stance [34]. Furthermore, BF activity remained unchanged during early swing phase knee flexion, while GM muscle load was reduced, indicating a heterogeneous distribution between the knee flexors. This can partly be explained by the fact that BF was already minimally engaged during knee flexion (< 10%), whereas GM, involved in both knee flexion and plantarflexion, seemed to have been relieved of their first function. To further investigate the effects of assistance on ankle joint muscles, it would also have been interesting to measure the activity of the antagonistic muscles (e.g., tibialis anterior). Even though the Keeogo did not assist the hip joint, it was largely impacted by knee assistance regarding muscle activation and kinematic as well. Muscle activation may also have been affected by reduced step length, gait speed, and reduced joint_RoM_. More specifically, hip flexion-extension_RoM_ during swing phase was reduced with the device. The use of the Keeogo, single joint assistance, may not only induce changes in assisted joint but biomechanical changes all over lower limb. This is in line with previous work using a device for ankle assistance that reported reduced knee extension range of motion [35]. The knee assistance did not change the knee joint shape, but the RoM timing was delayed, matching the start time of powered assistance. The knee extension angle was lower until it reached its peak, with delay, in comparison to gait cycle without the device (Fig. 4). The assistance caused a delay in knee flexion, which in turn delayed hip extension. The ankle plantarflexion peak occurred earlier with the Keeogo and exhibited greater variability. This may reduce the ability to generate forward propulsion through plantarflexion. The variability in peak occurrence may suggest that participants struggled to maintain propulsive function in the late stance phase. Interestingly, hip adduction also occurred sooner with the device. This means that participants adapted their kinematic timing by completing hip adduction earlier during the stance phase. This adjustment likely helped to compensate for the earlier ankle plantarflexion, allowing them to continue moving the leg forward, and contributed to decrease dynamic stability and reduced walking performance [36].

To the best of our knowledge, one study assessed sit-to-stand and stand-to-sit transitions with the Keeogo in stroke survivors; however, neither user perceptions nor kinematic measurements were reported [8]. At the group level, we showed that performances in STS30 was not significantly different with the Keeogo with large inter individual difference. The perceived dyspnea was lower, but as most participants did less repetitions with the device so that one cannot rule out that the overall workload might have been lower as well. The hip flexion-extension_RoM_ was reduced during sitting transitions when using the device. As observed in gait, the Keeogo changed the pattern of the hip while not inducing changes in knee and ankle joint kinematic. Lower hip flexion-extension_RoM_ was observed except for four participants. The latter were not the ones who increased their performance, suggesting that changes in hip kinematic might not be responsible for reduced performance during STS30. The temporal parameters also significantly changed with a longer time spent in sit-to-stand phase when using the device. The Keeogo requires participants to bend the trunk further with knee extension to trigger the assistance. This aspect is critical because it forces participants to bear the weight of the device in addition to its own. Altogether, longer sit-to-stand phase was due to longer time spent at the beginning of the phase when aiming to trigger the assistance. It is already known that the early phase of the sit-to-stand is longer in participants with NMD [37]. Interestingly, changes in gait parameters and kinematic parameters during STS30 affected only the Gmax muscle. The knee-powered assistance reduced Gmax workload, especially during the initial standing phase, by extending the knee after trunk flexion triggered the assistance. Secondly, at the beginning of the sitting phase, when the assistance slows knee flexion in an eccentric fashion, it allows participants to sit down more safely by limiting the risk of knee buckling. Unfortunately, the actual time-varying level of torque assistance that would have been helpful to determine whether participants with improved performance received greater knee assistance was unknown. A limitation of our work was the use of IMUs for kinematic assessment, which may be less accurate in populations with gait impairments [38]. To mitigate initialization errors, we reported only joint range of motion.

### Determinants of reduced gait performance when using the Keeogo

A model was developed to predict the 2MWT_perf_ based on outcomes from conditions without and with the device. The determinants highlighted by the model for reduced gait performance with the Keeogo in participants with NMD. Among the determinants, the knee extension and ankle dorsiflexion strength capacity were responsible for lower gait performances. Participants with greater strength in these muscle groups covered a longer distance than other participants, but their performance did not improve when assisted, suggesting that impaired strength capacity was not fully compensated by the device. Although the Keeogo (5.4 kg) is among the lightest powered knee exoskeletons currently available, its non-self-supporting design requires users to bear the entire load, which may offset potential benefits in individuals with severe weakness. This finding underscores the paradox between powered assistance and mechanical burden, suggesting that the device’s weight limited its compensatory potential. Gait parameters such as cadence was identified as a key determinant of gait performance. One may observe that for each participant, cadence was always lower when using the exoskeleton (Fig. 7). It seems likely that higher cadence in walking would participate to increase the distance covered with assistance. Off-note, higher cadence induced by the assistive device would potentially increase instability and the risk of falls in participants with NMD. In the gait cycle, the timing in hip extension and ankle dorsiflexion determined gait performance. The later the hip extension and ankle dorsiflexion occur, the lower will be the gait performance with the exoskeleton. Interestingly, no assistance was provided to these two joints, but participants with abilities to maintain ankle dorsiflexion before heel strike would benefit powered knee assistance from the Keeogo. Additionally, toe angle at toe-off time was determinant for 2MWT_perf_ which indicates an alteration of the propulsive phase caused by reduced peak angle at the end of the stance phase. The longer time spent in the support phase and the additional weight of the Keeogo weight prevented participants from propelling themselves as effectively as they were without the device. The assistance provided after mid-swing did not compensate for ankle dorsiflexor weakness, which is crucial for early ankle dorsiflexion. As a result, it could not prevent earlier and flatter foot contact at the early stage of the next gait cycle, potentially leading to a longer stance phase and reduced gait speed. Altogether, kinematic changes over unassisted joints were key components for longer stance phase which were responsible for reduced gait performance with the Keeogo and could be related to an increased search for stability to maintain safety. The prediction presented good performances, but the model was based on a small sample size, and it should be confirmed using larger datasets. Since all participants covered a shorter distance when using the device, one can consider these factors for future improvements of the device hardware and software.

### Determinants of performance changes in STS30 when using the Keeogo

A predictive model identified key factors from STS30 results without the device to estimate potential gains in STS30 performance with Keeogo. The determinants selected may be divided in two types: (i) level of muscle weakness, (ii) muscle workload without assistance. First, the model identified hip flexion and knee flexion strength capacity as determinants to predict the improvement in STS30_perf_. The greater the force production capacity of the hip and knee flexor muscles were, the more likely participants will be able to increase the repetitions in STS30. Hip flexors are essential in the early phase of the sit-to-stand during the flexion momentum. This phase begins with the initiation of the movement and ends just before the body leaves the chair [37]. The weakness of hip flexor muscles caused challenges to realize sit-to-stand task. In addition, the weight of the exoskeleton before assistance started highlights the importance of these muscles for improving STS30 performance. The knee flexors are crucial during the braking phase (at the end of sit-to-stand) by counteracting gravity with eccentric contraction [37]. They are thus essential for enhancing performance as they absorb the assistance provided by the exoskeleton and control the descent while sitting. Therefore, the decline in STS30 performance can be partly attributed to the inability to handle the overload during both the assisted phases (tolerated assistance level) and the non-assisted phases (managing the additional weight). Second, muscle activation of RF, VL, and Gmax during STS30 was identified as predictors of increased performances with assistance. Higher activation of the RF and Gmax muscles without the exoskeleton predicted improvement in STS30 performance. In contrast, greater VL activation correlated with a smaller change in performance. The RF muscle, used in both hip flexion and knee extension, plays a key role in sitting transfers while activated in the early phase of the sit-to-stand movement (0–20%) and in eccentric contraction to slow knee flexion preventing knee buckling (Fig. 4). The positive relationship between RF activation and performance improvement might be explained by its involvement in the early standing phase to trigger the assistance. The VL muscle, as a knee extensor, showed a negative relationship with performance improvement. In other words, the higher the VL muscle strength capacity was, the lesser would be the activation needed for knee extension, suggesting insufficient strength capacity would not benefit from the Keeogo’s assistance. Participants with lower Gmax muscle workload during STS30 would theoretically decrease their performance when using the device. As a hip extensor, the Gmax is not assisted by the device. The participant’s ability to straighten the trunk and finish the standing movement without assistance was key following inertial movement initiated from the assistance to benefit from the device. The model with the smaller number of determinants demonstrated better performances than the one using all the features. However, further investigation must be carried out with larger sample size and different groups of participants to validate these findings. Lower-limb strength capacity largely determined the performance of whether it was during walking or transfer’s task. Given that timing and force level are key parameters in providing assistance, it appears that this device did not allow participants to adequately compensate for strength impairments and was not well-suited to their movement compensations.

## Conclusion

This study aimed to evaluate the Keeogo exoskeleton’s effects on gait and transfer tasks in adults with neuromuscular diseases. Main findings indicate that while the device did not alter stability during quiet stance, it significantly reduced performance in gait and transfer tasks. Participants experienced reduced walking performances, with reduced muscle activation in some muscle groups and altered gait kinematics. The multivariate LASSO analysis complemented the descriptive findings by identifying key biomechanical and strength-related determinants associated with assisted performance. This integrative approach confirmed that knee extensor strength and ankle dorsiflexion range were primary predictors of walking performance, whereas hip control parameters were more influential during sit-to-stand transitions. These findings reinforce the interpretation that both muscle strength and coordination contribute to the individual response to exoskeletal assistance. Future developments should minimize weight and inertial load while optimizing assistance efficiency, particularly for users with severe and variably distributed muscle weakness. Addressing these factors will be critical to develop assistive devices that effectively meet the biomechanical needs of individuals with NMD. Finally, observing the long-term effects on gait and transfer, both with and without the Keeogo, would provide critical insights into its sustained impact.

## Supplementary Information

Supplementary material 1. Figure S1. Patient using the Keeogo device during a 10-m walk test conducted in a corridor setting ©Christophe Hargoues.

## Data Availability

The data supporting the findings of this study are available on request from the corresponding author. The data are not publicly available due to privacy or ethical restrictions.

## Abbreviations

NMD: Neuromuscular diseases
STS30: 30-Second sit-to-stand test
6MWT: 6-Minute walk test
MVC: Maximal voluntary contraction
EMG: Electromyography
SWAY: Postural static stability test
10mWT: 10-Meter walk test
2MWT: 2-Minute walk test
IMU: Inertial measurement unit
VAS: Verbal analog scale
NMQ: Nordic musculoskeletal questionnaire
SUS: System usability scale
Gmax: Gluteus maximus
BF: Biceps femoris
VL: Vastus lateralis
RF: Rectus femoris
GM: Gastrocnemius medialis
MVC_pred_: Predicted maximal voluntary contraction
2MWT_pred_: Predicted 2-min walk test distance
EMG_max_: Maximal electromyography value
EMG_peak_: Peak electromyography
EMG_AUC_: Area under the curve of electromyography
EMG_avg_: Electromyography average
EMG_SD_: Electromyography standard deviation
EMG_meanPF_: Electromyography mean power frequency
EMG_medianPF_: Electromyography median power frequency
JA_RoM_: Joint angle range of motion
JA_avg_: Joint angle average
JA_SD_: Joint angle standard deviation
ANOVA: Analysis of variance
LASSO: Least absolute shrinkage and selection operator
RMSE: Root mean squared error
MSE: Mean squared error
MAE: Mean absolute error

## References

[1] Mercuri E, Muntoni F. Muscular dystrophies. Lancet. 2013;381(9869):845–60. 23465426 10.1016/S0140-6736(12)61897-2

[2] Voet NBM. Exercise in neuromuscular disorders: a promising intervention. Acta Myol. 2019;38(4):207–14. 31970319 PMC6955632

[3] Horlings CG, Munneke M, Bickerstaffe A, Laverman L, Allum JH, Padberg GW, et al. Epidemiology and pathophysiology of falls in facioscapulohumeral disease. J Neurol Neurosurg Psychiatry. 2009;80(12):1357–63. 19546106 10.1136/jnnp.2009.173534

[4] Bryan GM, Franks PW, Song S, Reyes R, O’Donovan MP, Gregorczyk KN, et al. Optimized hip-knee-ankle exoskeleton assistance reduces the metabolic cost of walking with worn loads. J Neuroeng Rehabil. 2021;18(1):161. 34743714 10.1186/s12984-021-00955-8 PMC8572578

[5] Lakmazaheri A, Song S, Vuong BB, Biskner B, Kado DM, Collins SH. Optimizing exoskeleton assistance to improve walking speed and energy economy for older adults. J Neuroeng Rehabil. 2024;21(1):1. 38167151 10.1186/s12984-023-01287-5 PMC10763092

[6] Bach Baunsgaard C, Vig Nissen U, Katrin Brust A, Frotzler A, Ribeill C, Kalke YB, et al. Gait training after spinal cord injury: safety, feasibility and gait function following 8 weeks of training with the exoskeletons from Ekso Bionics. Spinal Cord. 2018;56(2):106–16. 29105657 10.1038/s41393-017-0013-7

[7] McGibbon CA, Sexton A, Jayaraman A, Deems-Dluhy S, Gryfe P, Novak A, et al. Evaluation of the Keeogo exoskeleton for assisting ambulatory activities in people with multiple sclerosis: an open-label, randomized, cross-over trial. J Neuroeng Rehabil. 2018;15(1):117. 30541585 10.1186/s12984-018-0468-6 PMC6291941

[8] McLeod JC, Ward SJ, Hicks AL. Evaluation of the Keeogo dermoskeleton. Disabil Rehabil Assist Technol. 2019;14(5):503–12. 29092649 10.1080/17483107.2017.1396624

[9] Rodríguez-Fernández A, Lobo-Prat J, Font-Llagunes JM. Systematic review on wearable lower-limb exoskeletons for gait training in neuromuscular impairments. J Neuroeng Rehabil. 2021;18(1):22. 33526065 10.1186/s12984-021-00815-5 PMC7852187

[10] Andersen LK, Knak KL, Witting N, Vissing J. Two- and 6-minute walk tests assess walking capability equally in neuromuscular diseases. Neurology. 2016;86(5):442–5. 26740680 10.1212/WNL.0000000000002332

[11] Voet NB, van der Kooi EL, van Engelen BG, Geurts AC. Strength training and aerobic exercise training for muscle disease. Cochrane Database Syst Rev. 2019;12(12):CD003907. 31808555 10.1002/14651858.CD003907.pub5 PMC6953420

[12] Feigean R, Afroun-Roca C, Guerrini C, Souchu J, Fer F, Benveniste O, et al. Key determinants of impaired gait performance in adults with neuromuscular diseases: a multiparametric and multimodal analysis. J Appl Physiol. 2025;138:1261–74. 40272844 10.1152/japplphysiol.00287.2024

[13] Maulet T, Cattagni T, Dubois F, Roche N, Laforet P, Bonnyaud C. Determinants and characterization of locomotion in adults with late-onset Pompe disease: new clinical biomarkers. J Neuromuscul Dis. 2023;10(5):963–76. 37545258 10.3233/JND-230060 PMC10578228

[14] Harbo T, Brincks J, Andersen H. Maximal isokinetic and isometric muscle strength of major muscle groups related to age, body mass, height, and sex in 178 healthy subjects. Eur J Appl Physiol. 2012;112(1):267–75. 21537927 10.1007/s00421-011-1975-3

[15] McAllister LS, Palombaro KM. Modified 30-second sit-to-stand test: reliability and validity in older adults unable to complete traditional sit-to-stand testing. J Geriatr Phys Ther. 2020;43(3):153–8. 30807554 10.1519/JPT.0000000000000227

[16] Raffegeau TE, Fawver B, Young WR, Williams AM, Lohse KR, Fino PC. The direction of postural threat alters balance control when standing at virtual elevation. Exp Brain Res. 2020;238(11):2653–63. 32944785 10.1007/s00221-020-05917-5 PMC8364805

[17] Martin-Martinez JP, Villafaina S, Collado-Mateo D, Fuentes-Garcia JP, Perez-Gomez J, Gusi N. Impact of cognitive tasks on biomechanical and kinematic parameters of gait in women with fibromyalgia: a cross-sectional study. Physiol Behav. 2020;227:113171. 32956683 10.1016/j.physbeh.2020.113171

[18] Bohannon RW, Wang YC, Gershon RC. Two-minute walk test performance by adults 18 to 85 years: normative values, reliability, and responsiveness. Arch Phys Med Rehabil. 2015;96(3):472–7. 25450135 10.1016/j.apmr.2014.10.006

[19] Crichton N. Visual analogue scale (VAS). J Clin Nurs. 2001;10(5):706.

[20] Bangor A, Kortum PT, Miller JT. An empirical evaluation of the System Usability Scale. Int J Hum Comput Interact. 2008;24(6):574–94.

[21] Kuorinka I, Jonsson B, Kilbom A, Vinterberg H, Biering-Sorensen F, Andersson G, et al. Standardised Nordic questionnaires for the analysis of musculoskeletal symptoms. Appl Ergon. 1987;18(3):233–7. 15676628 10.1016/0003-6870(87)90010-x

[22] Hermens HJ, Freriks B, Disselhorst-Klug C, Rau G. Development of recommendations for SEMG sensors and sensor placement procedures. J Electromyogr Kinesiol. 2000;10(5):361–74. 11018445 10.1016/s1050-6411(00)00027-4

[23] Hogrel JY, Payan CA, Ollivier G, Tanant V, Attarian S, Couillandre A, et al. Development of a French isometric strength normative database for adults using quantitative muscle testing. Arch Phys Med Rehabil. 2007;88(10):1289–97. 17908571 10.1016/j.apmr.2007.07.011

[24] De Luca CJ. Myoelectrical manifestations of localized muscular fatigue in humans. Crit Rev Biomed Eng. 1984;11(4):251–79. 6391814

[25] Desai R, Fritz NE, Muratori L, Hausdorff JM, Busse M, Quinn L. Evaluation of gait initiation using inertial sensors in Huntington’s Disease: insights into anticipatory postural adjustments and cognitive interference. Gait Posture. 2021;87:117–22. 33906090 10.1016/j.gaitpost.2021.04.021

[26] Kobayashi K, Umehara J, Pataky TC, Yagi M, Hirono T, Ueda Y, et al. Application of statistical parametric mapping for comparison of scapular kinematics and EMG. J Biomech. 2022;145:111357. 36395530 10.1016/j.jbiomech.2022.111357

[27] Meinshausen N, Bühlmann P. Stability selection. J R Stat Soc Ser B Stat Methodol. 2010;72(4):417–73.

[28] Tibshirani R. Regression shrinkage and selection via the Lasso. J R Stat Soc Series B Stat Methodol. 1996;58(1):267–88.

[29] Zou H, Hastie T. Regularization and variable selection via the elastic net. J R Stat Soc Ser B Stat Methodol. 2005;67(2):301–20.

[30] Bachasson D, Moraux A, Ollivier G, Decostre V, Ledoux I, Gidaro T, et al. Relationship between muscle impairments, postural stability, and gait parameters assessed with lower-trunk accelerometry in myotonic dystrophy type 1. Neuromuscul Disord. 2016;26(7):428–35. 27234310 10.1016/j.nmd.2016.05.009

[31] Park H, Branson D, Kim S, Warren A, Jacobson B, Petrova A, et al. Effect of armor and carrying load on body balance and leg muscle function. Gait Posture. 2014;39(1):430–5. 24021525 10.1016/j.gaitpost.2013.08.018

[32] McGibbon CA, Brandon SCE, Brookshaw M, Sexton A. Effects of an over-ground exoskeleton on external knee moments during stance phase of gait in healthy adults. Knee. 2017;24(5):977–93. 28760608 10.1016/j.knee.2017.04.004

[33] Swank C, Almutairi S, Wang-Price S, Gao F. Immediate kinematic and muscle activity changes after a single robotic exoskeleton walking session post-stroke. Top Stroke Rehabil. 2020;27(7):503–15. 32077382 10.1080/10749357.2020.1728954

[34] Wall-Scheffler CM, Chumanov E, Steudel-Numbers K, Heiderscheit B. Electromyography activity across gait and incline: the impact of muscular activity on human morphology. Am J Phys Anthropol. 2010;143(4):601–11. 20623603 10.1002/ajpa.21356 PMC3011859

[35] Gordon KE, Kinnaird CR, Ferris DP. Locomotor adaptation to a soleus EMG-controlled antagonistic exoskeleton. J Neurophysiol. 2013;109(7):1804–14. 23307949 10.1152/jn.01128.2011 PMC3628010

[36] Krebs DE, Robbins CE, Lavine L, Mann RW. Hip biomechanics during gait. J Orthop Sports Phys Ther. 1998;28(1):51–9. 9653690 10.2519/jospt.1998.28.1.51

[37] Sutcu G, Yalcin AI, Ayvat E, Kilinc OO, Ayvat F, Dogan M, et al. Electromyographic activity and kinematics of sit-to-stand in individuals with muscle disease. Neurol Sci. 2019;40(11):2311–8. 31222542 10.1007/s10072-019-03974-5

[38] Lallès AP, Moucheboeuf G, Doat E, Pillet H, Bonnet X. IMU calibration effect on lower limbs kinematics against optical motion capture in post-stroke gait. IRBM. 2024. 100873.

